# Towards infield, live plant phenotyping using a reduced-parameter CNN

**DOI:** 10.1007/s00138-019-01051-7

**Published:** 2019-12-17

**Authors:** John Atanbori, Andrew P. French, Tony P. Pridmore

**Affiliations:** 1 School of Computer Science, University of Nottingham, Nottingham, NG8 1BB UK; 2 School of Biosciences, University of Nottingham, Nottingham, LE12 5RD UK

**Keywords:** Pixel-wise segmentation for plant phenotyping, Lightweight deep convolutional neural networks, Separable convolutions, Singular value decomposition

## Abstract

There is an increase in consumption of agricultural produce as a result of the rapidly growing human population, particularly in developing nations. This has triggered high-quality plant phenotyping research to help with the breeding of high-yielding plants that can adapt to our continuously changing climate. Novel, low-cost, fully automated plant phenotyping systems, capable of infield deployment, are required to help identify quantitative plant phenotypes. The identification of quantitative plant phenotypes is a key challenge which relies heavily on the precise segmentation of plant images. Recently, the plant phenotyping community has started to use very deep convolutional neural networks (CNNs) to help tackle this fundamental problem. However, these very deep CNNs rely on some millions of model parameters and generate very large weight matrices, thus making them difficult to deploy infield on low-cost, resource-limited devices. We explore how to compress existing very deep CNNs for plant image segmentation, thus making them easily deployable infield and on mobile devices. In particular, we focus on applying these models to the pixel-wise segmentation of plants into multiple classes including background, a challenging problem in the plant phenotyping community. We combined two approaches (separable convolutions and SVD) to reduce model parameter numbers and weight matrices of these very deep CNN-based models. Using our combined method (separable convolution and SVD) reduced the weight matrix by up to 95% without affecting pixel-wise accuracy. These methods have been evaluated on two public plant datasets and one non-plant dataset to illustrate generality. We have successfully tested our models on a mobile device.

## Introduction

**Fig. 1:**
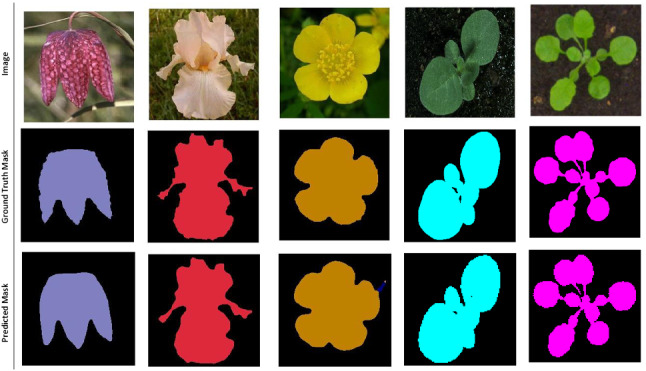
Flower and leaf images in the first row, their ground truth mask in the second row and the predicted CNN mask in the last row. The plants and flowers classes are predicted with a different colour indicating class. Images sources: The plant phenotyping and Oxford Flower datasets

The world population will reach 9.1 billion by 2050, about 34% higher than it is today. The UN Food and Agriculture Organisation (FAO) has estimated that in order to feed this larger and more urban population, food production must increase by 70% [5]. Plant phenotyping will play an important role in achieving this target. Plant phenotyping refers to a quantitative description of the plant’s anatomical, physiological and biochemical properties [34]. Traditionally, plant phenotyping is carried out by experts and involves manually measuring and recording plant traits, such as plant size and shape, number of leaves and flowers. High-quality, precise phenotyping of various plant traits can help improve yield under different climatic conditions (Fig. 1).

However, recently, image-based plant phenotyping has gained more attention due to its inherent merits in handling large-scale phenotyping: it is less tedious and error prone. In particular, image-based phenotyping techniques have been used in plant segmentation [1, 3] and leaf counting [1, 3, 11] and to automatically identify root and leaf tips [24]. Most of these approaches rely on visual plant trait identification, before measuring quantities that provide the data to discover high-yielding crops under different climatic conditions.

Today, deep convolutional neural networks have been used to phenotype plants in an attempt to gain much better accuracy [1, 21, 22, 25, 28]. In the computer vision community, these models have been shown to increase accuracy but at the expense of very many parameters (in millions) and expensive computations in the convolution layers (more multiplications and additions) [7, 17, 19, 39, 42]. Due to the number of model parameters, they are sometimes inefficient on low-cost, resource-limited devices.

Jin et al. [15], Wang et al. [35] and Iandola et al. [14] have attempted to reduce the computation time of CNNs, but the methods used by them were not applied to plant phenotyping. The objective of this research is to demonstrate how very deep CNN model parameters can drastically be reduced in number with very little reduction in pixel accuracy. In this paper we present the following new contributions. We have:

1. Formed ‘tiny’ models (models with the number of parameters drastically reduced) for pixel-wise segmentation by reducing the parameters of baseline very deep convolutional neural networks using separable convolution, without compromising pixel accuracy.
2. Demonstrated that the accuracy of these tiny models was as good as their baseline counterparts (un-compressed) on plant phenotyping datasets and a non-plant dataset.
3. Formed *very* tiny models (smaller weight matrix than the tiny models) using SVD and demonstrated on plant phenotyping datasets and a non-plant dataset that their pixel accuracy remains practically unaffected.
4. Evaluated the size of our ‘tiny’ models’ parameters with existing popular CNNs, demonstrating their potential for infield deployment.

The remainder of this paper is structured as follows. In Sect. 2, we review existing work that reduces model parameters and/or the weight matrix for devices with limited resources. In Sect. 3, we introduce the two public plant phenotyping datasets and the non-plant dataset used in our experiments and proceed in Sect. 4 to describe our methods used in compressing the baseline CNNs designed for pixel-wise segmentation. We describe our experimental set-up including a benchmark in Sect. 5. Then we proceed to present and discuss our results in Sect. 6 and conclude in Sect. 7

## Related work

Traditionally, plant phenotyping approaches using computer vision have looked at plant density estimation from RGB images [16, 18, 30] and counting leaves using a simple artificial neural network (ANN) or a support vector machine (SVM). However, these approaches are sometimes not fully automated and require some feature selection techniques to be applied prior to training classifiers. Minervini et al. [21] extracted dense SIFT descriptors from the green colour channel and quantised the SIFT space using k-means clustering to create a codebook for segmentation of plants. In a collation study [28], segmenting and counting leaves have also used traditional computer vision methods. The best results from these were based on super-pixel-based methods, watersheds and Chamfer matching. The results of these methods depend on the user fine-tuning parameters of the system and therefore may make them difficult for infield use. The super-pixel-based method needs the fine-tuning of five parameters, including those of canny edge detector in order to achieve good results. The watershed approach requires the use of morphological operations after plant segmentation to remove noise in the segmentation. This not only adds a step to the process but also requires additional parameter tuning step by the user.

More recent computer vision approaches to plant phenotyping are based on deep learning methods; these have been shown to perform better than the traditional methods [1]. Aich and Stavness [1] adopted the SegNet architecture and achieved better results on the dataset used in [21, 28]. The methods used by Aich and Stavness [1] have been used successfully by Aich et al. [3] in estimating phenotypic traits from wheat images and also in conjunction with global sum pooling [2] for counting wheat spikes accurately. Another plant phenotyping approach that uses deep learning achieved state-of-the-art automatic identification of ear base, leaf base, root tips, ear tips and leaf tips in wheat [24]. These deep learning approaches for plant phenotyping have been motivated by the recent successes in applying them to other fields for both segmenting and classification, some of which are considered in the remaining paragraphs of this section.

Long et al. [19] have popularised CNNs for dense predictions. The key features of their work are the $1\times 1$ convolution with the channel dimension equal to the number of classes being predicted, and a deconvolution layer used for bilinear up-sampling of the coarse outputs to a dense pixel output for prediction. Badrinarayanan et al. [7], however, showed that using the max-pooling indices to up-sample the coarse outputs can increase the pixel accuracy of the model. While Badrinarayanan et al. reported some improvements in pixel accuracies over the methods used by Long et al., their decoder had more parameters and was therefore less memory efficient. There have been other semantic segmentation networks [17, 40, 42], which achieved better pixel accuracies on similar datasets. However, these networks are very deep and thus have more parameters and use up more memory. The shortcomings of most convolutional neural networks lie within the convolutional layers and the fully connected layers. In the convolutional layer, the multiply and add operations are time-consuming and the fully connected layers also generate many parameters. It has been demonstrated by Yu et al. [38] that even though recognition accuracies of deep neural networks improve as the depth of a network increases, a large proportion of the parameters generated by these models contribute little to recognition and pixel accuracy.

Various attempts to reduce network size have focused on thinning the convolutional layer, reducing parameters in the fully connected layer of networks and compressing weight matrices generated by network models. While the first two focus on speeding up the training of models, the last focuses on testing. Reducing the number of parameters in a network can be achieved using a $1 \times 1$ convolution after $3 \times 3$ convolutions as in Inception [33] and ShuffleNet [41]. Depthwise separable convolutions have also been used in MobileNet [13] and Xception [8] to achieve this. ShuffleNet [41], however, used a combination of the two approaches. Since the vast majority of weight parameters reside in the fully connected layers, truncated SVD has been used in [9, 10, 31, 37] to reduce weight matrices in these layers. Denton et al. [9] and Girshick [10] demonstrated that using SVD speed up prediction while keeping accuracy within 1% of the original model.

The traditional approaches to plant phenotyping are usually semi-automated and thus not suitable for infield application. Recent developments in image-based plant phenotyping are based on state-of-the-art CNN methods. Even though these methods can be fully automated, they require significant storage and memory, thus making them unsuitable for deployment on low-cost devices (especially those with limited memory and processing power). Some current developments in CNNs aim to reduce their number of parameters, thus making them more efficient on low-cost devices but with some reduction in accuracy. This work, which builds on our previous [6], is based on this premise and, to the best of our knowledge, is one of the first applied to infield plant phenotyping.

Similar to our work, previous authors [4] have attempted to reduce the FCN and SegNet model parameters by replacing the deconvolution operation with sub-pixels [29] which introduced a negligible computational cost. However, our work is different from sub-pixel convolution since we focused on reducing parameters by replacing two-dimensional convolutions with two-dimensional separable convolution and then applying SVD.

## Datasets

**Fig. 2:**
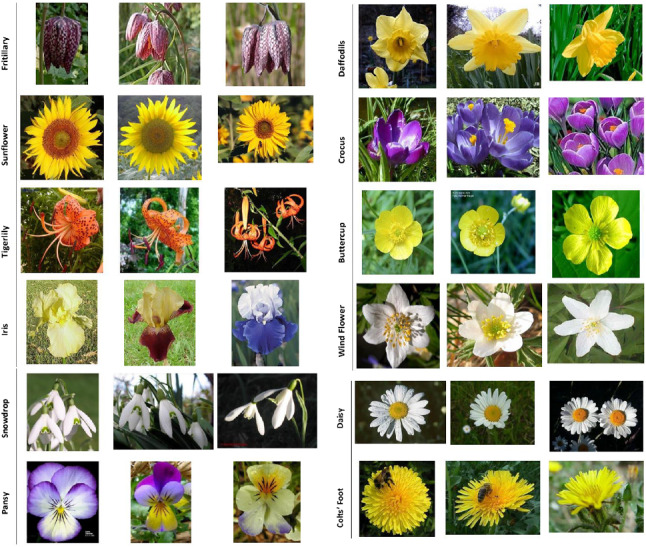
The Oxford flower dataset

**Fig. 3:**
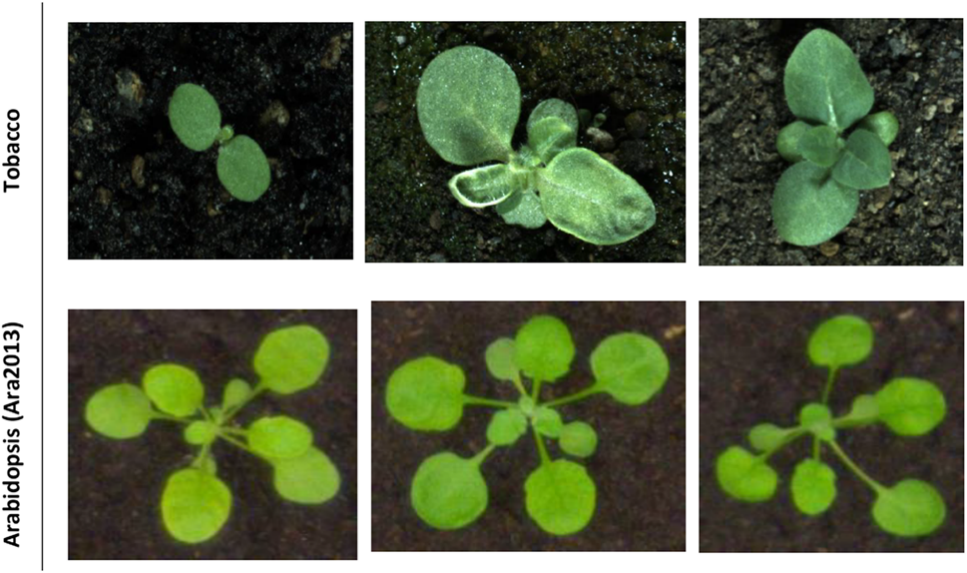
The plant phenotyping dataset

**Fig. 4:**
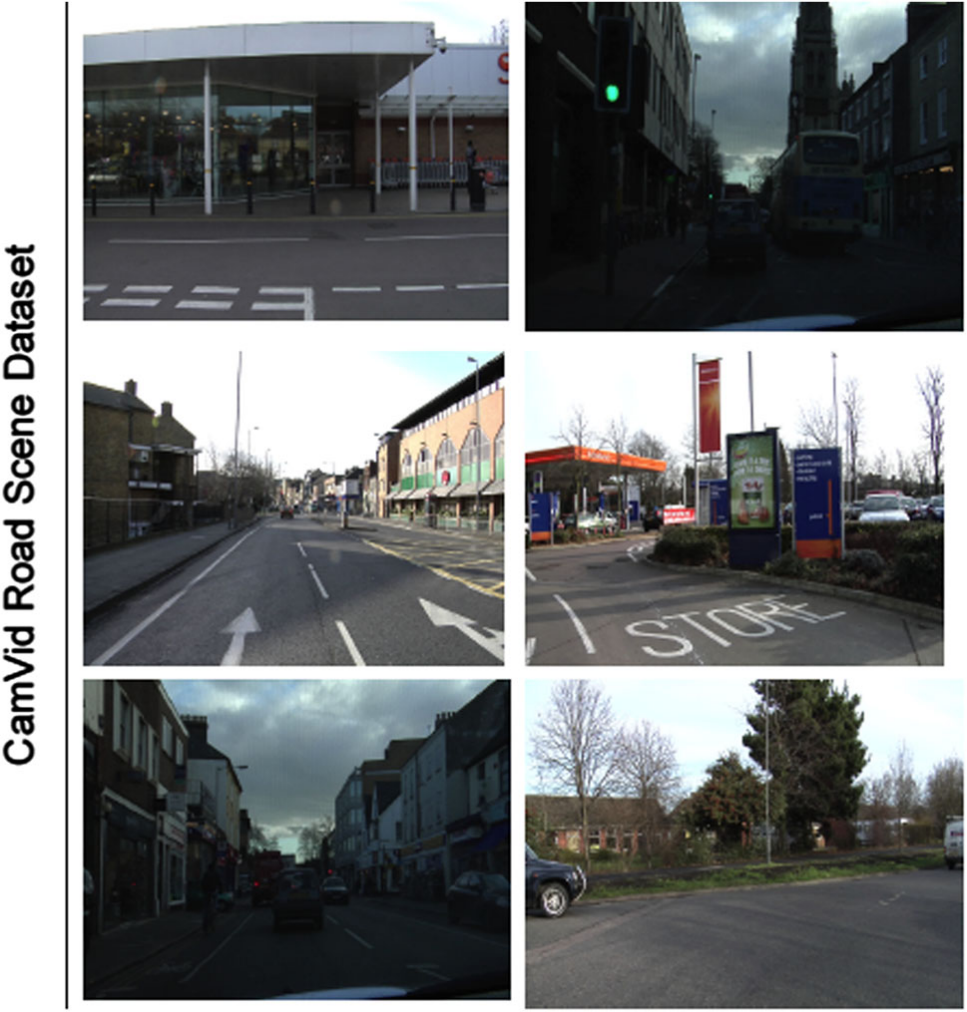
The CamVid dataset

We use two plant datasets: the Oxford flower dataset (Fig. 2) [23] and the CVPPP leaf segmentation challenge dataset also known as the plant phenotyping dataset (Fig. 3) [20, 21] and a non-plant dataset, the CamVid dataset (Fig. 4) [7] to perform our experiments.

The Oxford flower dataset has ground truth segmentation for most images. We use the same criteria as Nilsback and Zisserman [23] to form our segmentation dataset: flower classes that were under-sampled in the original dataset were removed. Following these criteria, five classes (Dandelion *Taraxacum*, lily of the valley *Convallaria majalis*, Cowslip *Primula veris*, Tulip *Tulipa* and Bluebell *Hyacinthoides non-scripta*) had insufficient images and were removed. This leaves 12 flower classes with a total of 753 images. Examples of images in this dataset are shown in Fig. 2.

The plant phenotyping dataset is a challenging dataset introduced in [21] and available online at http://www.plant-phenotyping.org/datasets. We used all 165 Arabidopsis images *(Arabidosis thaliana)* in the Ara2013 dataset and 62 tobacco *(Nicotiana tabacum)* images in the datasets. Examples of images in this dataset are shown in Fig. 3.

The CamVid dataset is a road scene understanding dataset with 367 training images, 101 validation images and 233 testing images of day and dusk scenes, available at http://mi.eng.cam.ac.uk/research/projects/VideoRec/CamVid/. The challenge is to segment 12 classes, including background, such as road, building, cars, pedestrians, signs, poles, and sidewalk. Examples of images in this dataset are shown in Fig. 4.

All plant datasets were first divided into ‘80/20’ for train/test; then the training data were further divided into ‘80/20’ for training/validation. We normalise all images by scaling RGB values to the range 0–1, before passing them to the deep neural networks. The RGB image annotations were first converted into a class label. For example, an RGB value of [255, 255, 0] belonging to class one is represented as [1, 1, 1], RGB value [255, 64, 64] belonging to class two is represented as [2,2,2], and so on. Finally, we converted the class labels into a binary class matrix (one-hot encoding) before passing them to our networks.

## Methods

We have reduced model parameters of three popular semantic segmentation networks (FCN, SegNet and Sub-Pixel) using the two methods detailed in this section. We used separable convolutions to reduce the model parameter number before training the network, and singular value decomposition to reduce weight matrix size after.

### Separable convolution

MobileNet [13], MobileNetV2 [27] and Xception [8] use separable convolution to reduce the model parameters. Separable convolution reduces the number of multiplications and additions in the convolutional operation, thus reducing the model’s weight matrix and speeding up the training and testing of large CNNs.

A 2D convolution can be defined as in Eq. 1.

1 $$\begin{aligned} y(m,n)=\sum _{i=0}^{k-1} \sum _{j=0}^{k-1} h(i,j)x(m-i,n-j) \end{aligned}$$

where *x* is the $(m \times n)$ matrix being convolved with a $(k \times k)$ kernel *h*. If the kernel *h* can be separated into two kernels, say $h_1$ of dimension $(m \times 1)$ and $h_2$ of dimension $(1 \times n)$, then the 2D convolution can be expressed as a 1D convolution as in Eq. 2.

2 $$\begin{aligned} y(m,n)=\sum _{i=0}^{k-1}h_1(i)\Bigg [ \sum _{j=0}^{k-1} h_2(j)x(m-i,n-j)\Bigg ] \end{aligned}$$

The 2D convolution requires $k \times k$ multiplications and additions. However, in the case of separable convolution, since the kernel is decomposed into two 1D kernels, the required multiplications and additions are reduced to $k+k$, thus reducing the number of model parameters.

**Fig. 5:**
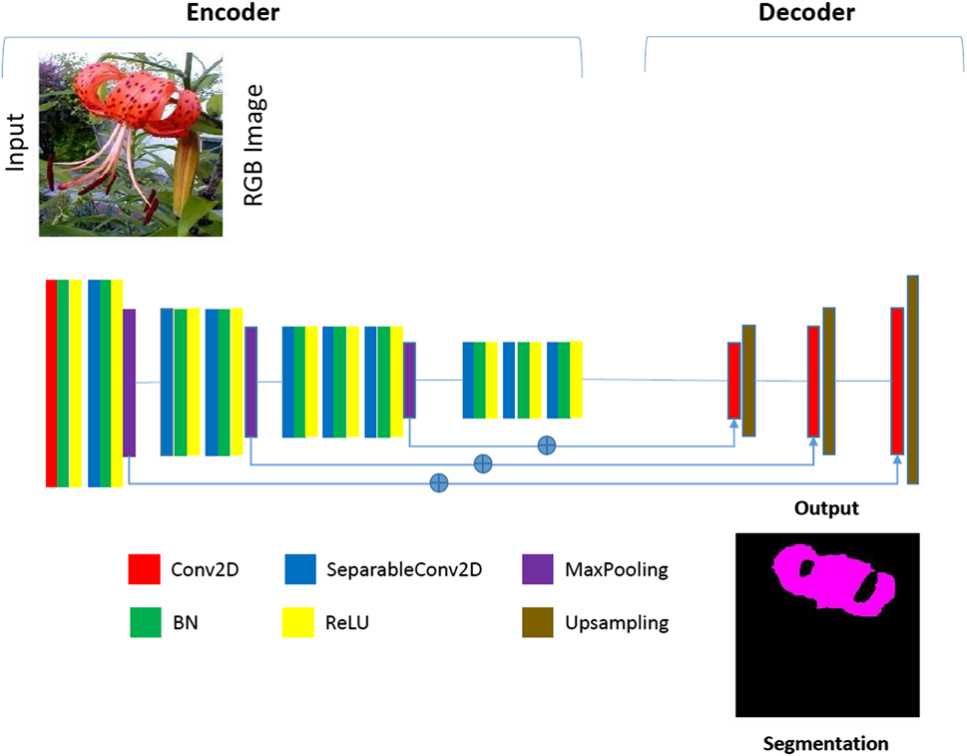
Architecture of our Tiny-FCN. This is a typical VGG-19 architecture with only four blocks. The building blocks are comprised of a 2D convolution (Conv2D), 2D seperable convolution (SeparableConv2D), batch normalisation (BN), a ReLU activation, max-pooling and up-sampling

**Fig. 6:**
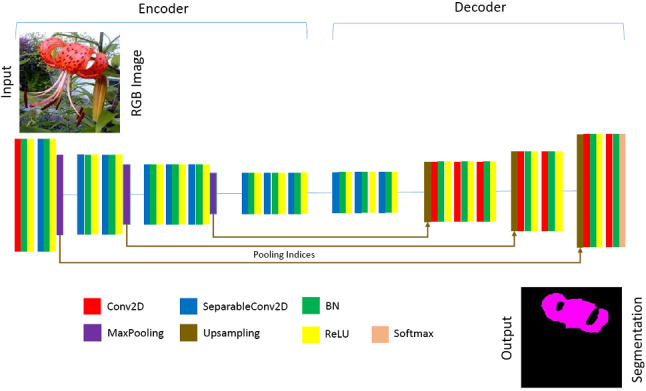
Architecture of our Tiny-SegNet. This is a typical VGG-19 architecture with only four blocks. The building blocks are comprised of a 2D convolution (Conv2D), 2D seperable convolution (SeparableConv2D), batch normalisation (BN), ReLU and softmax activations, max-pooling and up-sampling

We converted the 2D convolutions in the baseline semantic segmentation networks (FCN, SegNet and Sub-Pixel) into separable versions. For SegNet, the convolutional layers in both the encoder and decoders were made separable. However, with FCN and Sub-Pixel only the encoders were separable, as the decoder had few or no parameters. We then applied batch normalisation and ReLU activations to the separable convolutions. It is important to note that the first convolution layer of each network was not separated, as this holds important high-detail features. The reduced architectures are illustrated in Figs. 5 and 6

### Singular value decomposition

Singular value decomposition, which has been successfully applied to image compression [26], can be used to reduce the size of weight matrices [9, 10, 37]. Assuming $\mathbf {W} \in \mathbb {R}^{m \times n}$ is the weight matrix from the separable convolutions model, then the singular value decomposition of matrix *W* can be factorised into the form shown in Eq. 3.

3 $$\begin{aligned} W = U \cdot S \cdot V^T \end{aligned}$$

where $\mathbf {U} \in \mathbb {R}^{m \times n}$ is an $m \times n$ left-singular vector, $\mathbf {V} \in \mathbb {R}^{n \times n}$ is an $n \times n$ right-singular vector and $\mathbf {S} \in \mathbb {R}^{n \times n}$ is an $n \times n$ rectangular diagonal matrix called the singular values of the weight matrix *W*. Then assuming diagonals of $S = \{d_{(1,1)}, d_{(2,2)}, d_{(3,3)}..., d_{(n,n)}\}$ and $\{d_{(1,1)} \ge d_{(2,2)} \ge d_{(3,3)} \ge ... \ge d_{(n,n)} \ge 0\}$, we can reconstruct a new matrix $W^\prime$ as in Eq. 4

4 $$\begin{aligned} W^\prime = U^\prime \cdot S^\prime \cdot {V^T}^\prime \end{aligned}$$

where $$\mathbf {U}^{\prime } \in \mathbb {R}^{m \times k}$$, $$\mathbf {V}^{{\mathbf {T}}^{\prime }} \in \mathbb {R}^{k \times n}$$ and $$\mathbf {S}^{\prime } \in \mathbb {R}^{k \times k}.$$

$\mathbf {W}^{\prime } \in \mathbb {R}^{m \times n}$ is the reconstructed weight matrix, which has the same dimensions as *W*. It is important to note that $W^\prime$ was reconstructed with the first *k* singular values of *S* and $k = min(m, n)$. Selecting *k* in this way reduces the size of the weight matrix.

We compressed the weight matrices generated by the separable convolution models (which we call Tiny-FCN, Tiny-SegNet and Tiny-Sub-Pixel) to form a very tiny model (which we call Very-Tiny-FCN, Very-Tiny-SegNet and Very-Tiny-Sub-Pixel, respectively) using the SVD approach presented in this section. In both models, we skipped the first three blocks and only applied SVD to the remainder, as this will ensure that high-detail features are not lost and thus not drastically reduce the model’s performance, as the first three blocks already have a small number of parameters.

## Experiments

For our evaluation, we used the three datasets detailed in Sect. 3 to perform the following experiments. We produce:

- Pixel-wise segmentation into classes using the original semantic segmentation networks (FCN, SegNet and Sub-Pixel)
- Pixel-wise segmentation into classes using our tiny models, Tiny-FCN, Tiny-SegNet and Tiny-Sub-Pixel, which is made up of only separable convolutions.
- Pixel-wise segmentation into classes using our very tiny models, Very-Tiny-FCN, Very-Tiny-SegNet and Very-Tiny-Sub-Pixel, which is made up of separable convolutions and SVD.
- Background and foreground segmentation (two classes) of the Oxford flower dataset to help further evaluate the models on smaller datasets and to show that the baseline models performed better with fewer classes.

### Set-up

We perform all our experiments using the VGG-16 style architecture but without the last block, known as VGG-16 Basic, as recommended by Badrinarayanan et al. [7] when evaluating SegNet, FCN and Sub-Pixel. The convolutional layers in each model’s encoder were followed by batch normalisation and ReLU activation layers. Except for the last, we placed a max-pooling layer at the end of each encoder block.

The FCN architectures (including the ‘Tiny’ versions) used the FCN-8 decoder style as described in [19]. Since the FCN’s decoder had fewer parameters, we did not perform separable convolutions on them. However, with the exception of the first layer, all convolutional layers of the Tiny-FCN encoder were converted into separable convolutions and then each was followed by a batch normalisation and ReLU activation layers. The set-up of the Sub-Pixel architecture is similar to the FCN but its decoder is made of sub-pixel convolution, which generates no parameters.

The SegNet used same settings as in [7] and we used the max-pooling indices for up-sampling. Tiny-SegNet’s encoder used a similar set-up as the Tiny-FCNs. Similarly, apart from the first layers, all convolutional layers were converted into a separable convolution and followed with a batch normalisation and ReLU activation layers. Unlike Tiny-FCN, we applied separable convolutions to all convolutional layers of SegNet decoder and followed them by batch normalisation and ReLU activation layers, to form our Tiny-SegNet model.

Training of the CNN models was performed on a Linux server with three GeForce GTX TITAN X GPUs (12 GB memory each). The models were implemented using Python 3.5.3 and Keras 2.0.6 with Tensorflow backend and were tested on a windows 10 computer with 64 GB RAM and a 3.6 GHz processor. We also developed a mobile app to test capabilities of our tiny models using Android studio 3.1.2 on windows and tested it using a 1) Samsung Galaxy J1 mobile phone running Android 4.4 and 2) Google Nexus 5x mobile phone emulator running Android 8.1.

### Benchmarks

For benchmarking, we compared with the baseline models (FCN, SegNet and Sub-Pixel) on the three datasets, the Oxford flower dataset (http://www.robots.ox.ac.uk/~vgg/data/flowers/17/index.html), the plant phenotyping dataset (http://www.plant-phenotyping.org/datasets) and the CamVid dataset (http://mi.eng.cam.ac.uk/research/projects/VideoRec/CamVid/), We compare the results to our ‘tiny’ models (Tiny-FCN, Very-Tiny-FCN, Tiny-SegNet and Very-Tiny-SegNet). In particular, we evaluated the following:

- Number of parameters per our model versus the baseline deep CNN models.
- Size of weight matrix per our model versus the baseline deep CNN models
- Accuracy (pixel accuracy, mean IoU, precision and recall) per model
- Average processing time using three devices for segmentation.

We also performed background and foreground (two-class) segmentation with the Oxford flower dataset to evaluate the performance of the FCN, SegNet and Sub-Pixel models with the multi-class segmentation. We tested the tiny models on two types of mobile device: Google Nexus 5x and Samsung Galaxy J1 smartphone. The Samsung Galaxy J1 was used also for real-time infield segmentation, to show that the tiny models work on mobile devices infield. Finally, we compare tiny and very-tiny model parameters to some popular existing models (see Table 8) that have used some form of parameter and or weight matrix reduction technique.

For all models, we set the number of epochs to 200 with a batch size of 6. We use categorical cross-entropy loss as the objective function for training the network and an Adam optimiser with an initial learning rate of 0.001. We then reduced the learning rate by a factor of 10 whenever training plateaus for more than 10 epochs. The input images were all resized to $(224 \times 224)$ since most input images were approximately this size, and also to help avoid a fractional output size that may result from the max-poolings in the network. We did not apply data augmentation as there were no problems of overfitting and the performance of the models was good.

Due to the large variations in the number of pixels in each class as per the training samples, we weighted the loss differently based on the true class (known as class balancing). We applied median frequency balancing, which is the ratio of median class frequency computed on the entire training samples divided by the class frequency. The implication of this is that larger classes in the training set are given less weight, while smaller ones are given more.

**Fig. 7:**
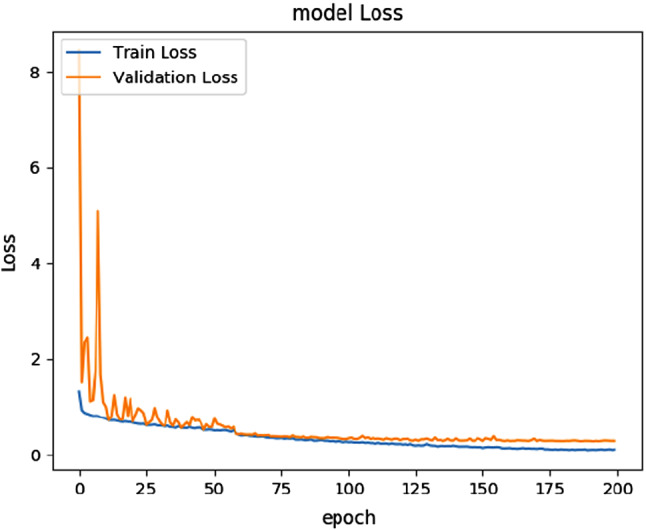
The training and validation loss versus epochs’ curves for the flower dataset based on the SegNet model

**Fig. 8:**
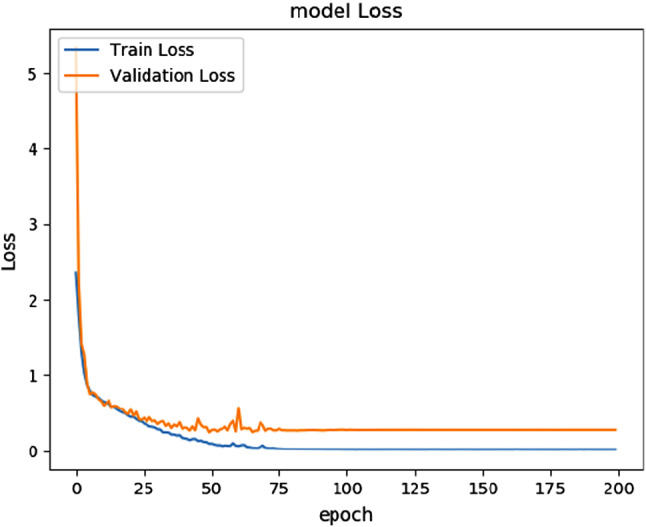
The training and validation loss versus epochs curves for the flower dataset based on the Tiny-Sub-Pixel model

Finally, we ensured that the baseline models and tiny models neither over- nor underfit by monitoring the training and validation losses. Figures 7 and 8 show the loss curves for SegNet and Tiny-Sub-Pixel on the flower dataset for 200 epochs. Both figures represent a drop in training and validation error as the number of epochs increases, which indicates that the networks are learning from the data that are given as input and not overfitting or underfitting. Similar pattern curves occurred for all the other models, which can be downloaded from this section’s footnote^1^.

### Metrics used

We report four metrics from common pixel-wise segmentation evaluations that are variations on pixel accuracy and region intersection over union (IoU), where $n_{ij}$ is the number of pixels of class *i* predicted to belong to class *j*, $n_{ji}$ is the number of pixels of class *j* predicted to belong to class *i* and *c* is the total number of classes.

- **Pixel accuracy**: This tells us about the overall effectiveness of the classifier and is defined in Eq. 5.
  5 $$\begin{aligned} \frac{\sum _{i=1}^{c}n_{ii}}{\sum _{i=1}^{c} (\sum _{j=1}^{c}n_{ij}) } \end{aligned}$$
- **Mean IoU**: This compares the similarity and diversity of the complete sample set and is defined in Eq. 6:
  6 $$\begin{aligned} \frac{1}{c}*\sum _{i=1}^{c}\frac{n_{ii}}{\sum _{j=1}^{c}n_{ij} + (\sum _{j=1}^{c}n_{ji}) - n_{ii} } \end{aligned}$$
- **Average Precision**: This tells us about the class agreement of the data labels with the positive labels given by the classifier and is defined in Eq. 7.
  7 $$\begin{aligned} \frac{1}{c}*\sum _{i=1}^{c}\frac{n_{ii}}{\sum _{j=1}^{c}n_{ji}} \end{aligned}$$
- **Average Recall**: This is the effectiveness of classifier to identify positive labels and is defined in Eq. 8.
  8 $$\begin{aligned} \frac{1}{c}*\sum _{i=1}^{c}\frac{n_{ii}}{\sum _{j=1}^{c}n_{ij}} \end{aligned}$$

## Results

Table 1 shows the results of parameter reduction when we applied only separable convolutions (Tiny-FCN, Tiny-SegNet and Tiny-Sub-Pixel models) and when we combined separable convolutions and SVD (our Very-Tiny-FCN, Very-Tiny-SegNet and Very-Tiny-Sub-Pixel models). The highlighted rows show data for the existing pixel-wise segmentation models, which we used as our baseline models.

**Table 1:** Model parameters and size of weight matrices on disc for all models used in our experiments

	Parameters		Weight matrix	
	#	Reduction (%)	Size on disc (MB)	Storage savings (%)
**FCN**	**7,647,950**	–	**87.6**	–
Tiny-FCN	885,528	88.42	10.2	88.36
Very-Tiny-FCN	885,528	88.42	3.51	95.99
**SegNet**	**17,649,795**	–	**202.0**	–
Tiny-SegNet	2,034,499	88.47	23.4	88.42
Very-Tiny-SegNet	2,034,499	88.47	7.96	96.06
**Sub-Pixel**	**7,646,043**	–	**88.0**	–
Tiny-Sub-Pixel	881,142	88.48	10.9	87.6
Very-Tiny-Sub-Pixel	881,142	88.48	3.6	95.9

The baseline models have been highlighted in bold

The models compressed with only separable convolution achieved a little above 88% in storage space savings. However, our models compressed using both separable convolution and SVD had the most storage space savings.

**Table 2:** Accuracies for both original and tiny models based on the plant phenotyping dataset

	Precision (%)	Recall (%)	Pixel accuracy (%)	Mean IoU (%)
**FCN**	98.59	98.57	98.58	95.49
Tiny-FCN	98.45	98.44	98.45	95.47
Very-Tiny-FCN	98.45	98.44	98.45	95.47
SegNet	98.27	98.20	98.23	94.82
Tiny-SegNet	98.09	98.03	98.06	94.07
Very-Tiny-SegNet	98.09	98.03	98.06	94.07
**Sub-Pixel**	**98**.**68**	**98**.**62**	**98**.**65**	**96**.**20**
Tiny-Sub-Pixel	98.73	98.56	98.65	96.18
Very-Tiny-Sub-Pixel	98.73	98.56	98.65	96.18

Plants were segmented into three classes

Table 2 shows the accuracies of the baseline deep CNN models versus their tiny counterparts. These results are based on segmenting the test samples of the plant phenotyping dataset into three classes (background, Tobacco *(Nicotiana tabacum)* and Arabidopsis *(Arabidopsis thaliana)* plants). The best performing models based on this dataset are the FCNs and Sub-Pixel, which outperformed the SegNet models by almost 1% based on mean IoU. The difference in mean IoU between the tiny models and original deep CNN counterpart is less than 0.75% and 0.02% for the SegNet and FCN, respectively, which shows that our compressed FCN and SegNet models are comparable to the original deep CNN.

**Table 3:** Accuracies for both original and tiny models based on the Oxford flower dataset

	Precision (%)	Recall (%)	Pixel accuracy (%)	Mean IoU (%)
FCN	94.98	94.02	94.38	72.73
Tiny-FCN	94.08	93.29	93.57	72.38
Very-Tiny-FCN	94.08	93.29	93.57	72.38
**SegNet**	**95**.**08**	**94**.**26**	**94**.**41**	**74**.**51**
Tiny-SegNet	94.46	94.06	94.20	74.50
Very-Tiny-SegNet	94.46	94.06	94.20	74.50
Sub-Pixel	94.21	94.04	94.04	72.18
Tiny-Sub-Pixel	93.81	93.60	93.72	71.92
Very-Tiny-Sub-Pixel	93.81	93.60	93.72	71.92

The flowers were segmented into 13 classes

In Table 3, we present the accuracies of the baseline deep CNN models versus their tiny counterparts on the test samples in the Oxford flower dataset, segmenting into 13 classes including the background. The results show the SegNet models to perform better than the FCN based on all the evaluation metrics. The baseline SegNet and FCN models outperformed their tiny counterparts by less than 0.01% and 0.35% based on mean IoU, respectively. This shows the tiny models to be comparable to the baselines used in our experiments.

**Table 4:** Accuracies for both original and tiny models based on the CamVid dataset

	Precision (%)	Recall (%)	Pixel accuracy (%)	Mean IoU (%)
**FCN**	**93**.**01**	**92**.**73**	**92**.**25**	**55**.**89**
Tiny-FCN	90.73	88.88	88.98	51.59
Very-Tiny-FCN	90.73	88.88	88.98	51.59
SegNet	92.59	89.75	90.85	54.48
Tiny-SegNet	90.87	89.77	89.80	53.69
Very-Tiny-SegNet	90.87	89.77	89.80	53.69
Sub-Pixel	93.51	88.72	88.85	51.89
Tiny-Sub-Pixel	90.53	88.12	88.28	51.77
Very-Tiny-Sub-Pixel	90.53	88.12	88.28	51.77

The road scenes were segmented into 12 classes (including the background)

To further investigate the results and illustrate generality of our multi-class segmentation, we trained all models on the CamVid dataset, which is of similar size as our multi-class flower dataset but for a different problem domain (road scenes instead of plants). Table 4 shows the accuracies of baseline deep CNN models versus their tiny counterparts based on segmenting test samples of this dataset into 12 classes including the background. We observe an interesting result, which this time shows the FCN models to outperform the SegNet models by approximately 1%. Furthermore, the very deep FCN and SegNet models outperformed their tiny counterparts by approximately 3% and 0.8% mean IoU, respectively.

**Table 5:** Accuracies for both original and tiny models based on the Oxford 17 flower dataset

	Precision (%)	Recall (%)	Pixel accuracy (%)	Mean IoU (%)
FCN	97.10	97.10	97.10	93.29
Tiny-FCN	96.80	96.80	96.80	92.64
Very-Tiny-FCN	96.80	96.80	96.80	92.64
**SegNet**	**97**.**27**	**97**.**27**	**97**.**27**	**93**.**65**
Tiny-SegNet	97.08	97.08	97.08	93.24
Very-Tiny-SegNet	97.08	97.08	97.08	93.24
Sub-Pixel	97.18	97.18	97.18	93.47
Sub-Pixel	96.92	96.92	96.92	92.87
Tiny-Sub-Pixel	96.92	96.92	96.92	92.87

The flowers were segmented into two classes (flowers and background)

We segmented the test samples in the Oxford flower dataset into just two classes (background and flower) using all baseline and tiny models. We present the results in Table 5. The result shows the baseline deep CNN models, and their tiny counterparts, to perform better on this dataset with two classes. The two-class problem outperformed the 13-class problem by approximately 19% based on mean IOU alone. SegNet outperformed FCN on this problem domain by a very narrow margin. Furthermore, even though SegNet was the best performing model, the other models remain comparable.

**Fig. 9:**
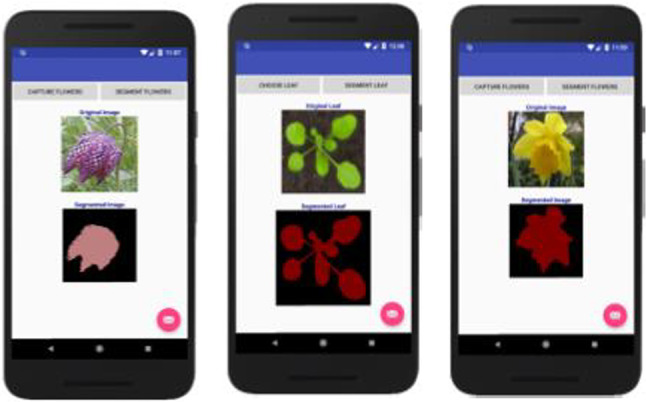
Mobile test results using Google Nexus 5X emulators. From left to right: Flower segmentation into 13 classes, leaf segmentation into 3 classes and flowers segmentation into 2 classes (foreground and background)

**Fig. 10:**
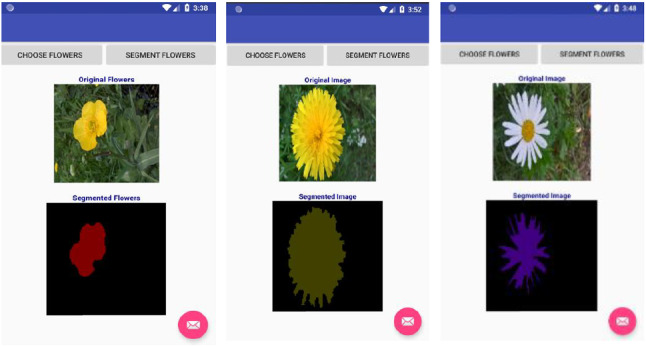
Real-time infield test on Samsung Galaxy J1 smart phone. This was performed only for the flowers dataset

**Fig. 11:**
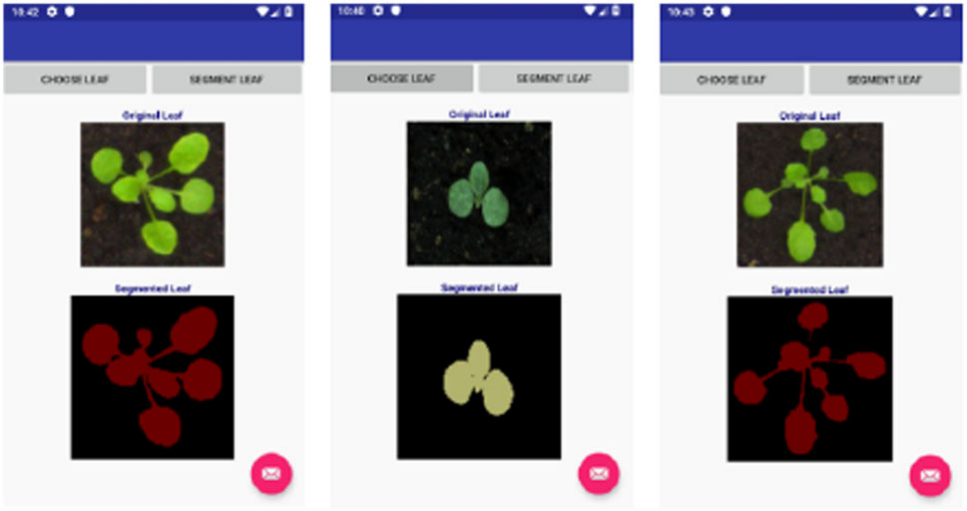
Segmenting leaf data collected from the internet on the Samsung Galaxy J1 smart phone

Finally, we developed mobile applications using Android studio to show that our tiny models can run well on these devices compared with the baseline models. We tested the applications on a Google Nexus 5X emulator and Samsung Galaxy J1 smartphone. Figures 9 and 10 show the results of segmentation on the flowers dataset for Google Nexus 5X and Samsung Galaxy J1, respectively. We have also included Fig.  11, which shows the results of using the Samsung Galaxy J1 for segmenting leaf images collected from the internet. The Samsung Galaxy J1 has been used infield successfully to segment flowers, while the Google Nexus 5X was only emulated with Android studio. The average processing speed was tested for segmenting flowers and leaves only. We used the data captured infield with the Samsung Galaxy J1 to test the flower mobile application, and a set of images collected from the web to test the leaf mobile application, and then discuss these results in Sect. 6.1.

**Table 6:** Average processing speed in seconds for segmenting a leaf and a flower into two or 13 classes using the **tiny models**

	Windows	Nexus 5x	Samsung J1
Tiny-FCN			
Flower-2 classes	$0.10 \pm 0.01$	$0.18 \pm 0.03$	$3.32 \pm 0.24$
Flower-13 classes	$0.11 \pm 0.02$	$0.19 \pm 0.03$	$3.95 \pm 0.30$
Leaf	$0.12 \pm 0.02$	$0.20 \pm 0.05$	$3.91 \pm 0.14$
Tiny-SegNet			
Flower-2 classes	$0.16 \pm 0.02$	$0.21 \pm 0.03$	$5.32 \pm 0.14$
Flower-13 classes	$0.18 \pm 0.01$	$0.27 \pm 0.06$	$6.13 \pm 0.23$
Leaf	$0.17 \pm 0.07$	$0.31 \pm 0.07$	$6.42 \pm 0.13$
Tiny-Sub-Pixel			
Flower-2 classes	$0.13 \pm 0.04$	$0.18 \pm 0.05$	$3.92 \pm 0.31$
Flower-13 classes	$0.14 \pm 0.06$	$0.18 \pm 0.04$	$4.21 \pm 0.40$
Leaf	$0.14 \pm 0.04$	$0.19 \pm 0.07$	$4.37 \pm 0.22$

These have been tested on three devices (Windows 10 computer, Google Nexus 5x emulator and Samsung J1 mobile). These were computed using 15 test flower and leaf images. The average processing speed shows plus/minus standard deviation

We noted that the smaller parameter models were faster in segmenting both flowers and leaves (see Tables 6 and 7). As expected, the ‘tiny’ models process faster than the baseline model since they have fewer parameters. The FCN and Sub-Pixel-based models with only 0.9 million parameters segment flowers and leaves nearly 2 s faster than the SegNet models for 13 classes (see Table 7).

The average processing speed of segmenting a single flower infield with the Samsung J1 mobile phone is 3.32 and 3.95 s for the two- and 13-class problems, respectively. Segmenting the same images using a Windows computer or a Google Nexus 5x mobile phone emulator is faster due to their considerably higher processing power. Considering that the Samsung J1 only runs an Android 4.4.4 compared to Android 8.1 on Google Nexus 8.1 further justifies the results.

Both baseline FCN and Sub-Pixel models have 7.6 million parameters and take approximately 3 s to segment a flower or a leaf on a windows 10 computer, 5 s on the Google Nexus 5x and 23 s on the Samsung J1 mobile phone. The baseline SegNet model is the slowest to process a flower or leaf, even though this takes approximately 7 and 8 s on windows 10 and Google Nexus 5x, respectively. When segmenting with SegNet model (17.5 million parameters), the application crashes due to the large number of parameters and the low processing power of this device.

**Table 7:** Average Processing speed in seconds for segmenting a leaf and a flower into two or 13 classes using the **Baseline models**

	Windows	Nexus 5x	Samsung J1
FCN			
Flower-2 classes	$2.59 \pm 0.14$	$4.02 \pm 0.17$	$22.41 \pm 0.82$
Flower-13 classes	$2.73 \pm 0.12$	$4.10 \pm 0.10$	$23.05 \pm 0.58$
Leaf	$2.37 \pm 0.07$	$3.91 \pm 0.12$	$22.90 \pm 0.52$
SegNet			
Flower-2 classes	$6.73 \pm 0.14$	$7.95 \pm 0.23$	–
Flower-13 classes	$6.93 \pm 0.21$	$8.01 \pm 0.15$	–
Leaf	$6.64 \pm 0.16$	$7.75 \pm 0.24$	–
Sub-Pixel			
Flower-2 classes	$2.49 \pm 0.10$	$3.96 \pm 0.08$	$21.21 \pm 059$
Flower-13 classes	$2.94 \pm 0.13$	$4.03 \pm 0.11$	$22.01 \pm 071$
Leaf	$2.91 \pm 0.09$	$3.89 \pm 0.11$	$21.73 \pm 0.61$

These have been tested on three devices (Windows 10 computer, Google Nexus 5x emulator and Samsung J1 mobile). These were computed using 15 test flower and leaf images. The average processing speed shows plus/minus standard deviation

### Discussion

We present in Table 8 the number of parameters in millions for some popular segmentation models. The top models were our Tiny-FCN, Very-Tiny-FCN, Tiny-Sub-Pixel and Very-Tiny-Sub-Pixel models, which all had less than a million parameters. The Very-Tiny-Sub-Pixel had the smallest number of parameters when compared to the nearest thousand. The replacement of the decoders with sub-pixel convolution made this possible. The other models used some form of parameter reduction techniques while preserving the accuracy of the model. SqueezeNet (1.3 million) was the next model reduced in parameters followed by our Tiny-SegNet and Very-Tiny-SegNet models and then MobileNet, which were all designed for mobile platforms.

**Table 8:** Comparing parameters of some popular models with ours

Model	Parameters (Millions)
Tiny-FCN (Ours)	0.9
Very-Tiny-FCN (Ours)	0.9
Tiny-Sub-Pixel (Ours)	0.9
Very-Tiny-Sub-Pixel (Ours)	0.9
SqueezeNet [14]	1.3
Tiny-SegNet (Ours)	2.0
Very-Tiny-SegNet (Ours)	2.0
MobileNet[13]	4.2
GoogleNet [32]	6.8
Sub-Pixel [4]	7.6
FCN (VGG-16 Basic) [19]	7.6
VGG-16 Compressed [12]	11.3
AlexNet - QCNN [36]	12.6
SegNet (VGG-16 Basic)[7]	17.5
Xception [8]	22.9
Inception V3 [33]	23.2
SVD [9]	47.6

The number of parameters is in millions

Howard et al. [13] reported that reducing CNN model parameters using separable convolution usually reduces the accuracy of the network. The experiments we performed also confirm this finding. We observed that with a careful reduction in the number of parameters in the baseline deep CNN model, accuracies are comparable. We noted that when using separable convolutions to reduce model parameters, a good practice is not to apply them to convolutional layers with a smaller number of parameters. For example, we only applied separable convolutions to the FCN encoder but not the decoder. Due to this, Howard et al. had used a parameter called depth multiplier which controls the number of channels generated as output ($output\_channels = input\_channels * depth\_multiplier$). Thus, using a smaller $depth\_multiplier$ shrinks the model parameters even further but at the expense of accuracy. We preferred to work with a depth multiplier of one, as this produces good results when separable convolutions are applied to large parameter generating convolutional layers.

Furthermore, caution is needed when using singular value decomposition to reduce model weight matrices. Some works have reported a drop in accuracy when SVD is applied [9, 10, 31, 37]. Our results show that applying SVD correctly further decreases the size of weight matrices while preserving pixel accuracies.

Skipping the first three convolutional layers, we apply SVD (with $k=4$) to all other convolutional layers to reconstruct the models’ weight matrices. This careful application of SVD not only reduced the size of the model on disc but also resulted in comparable pixel accuracies to the state-of-the-art non-compressed counterparts.

**Fig. 12:**
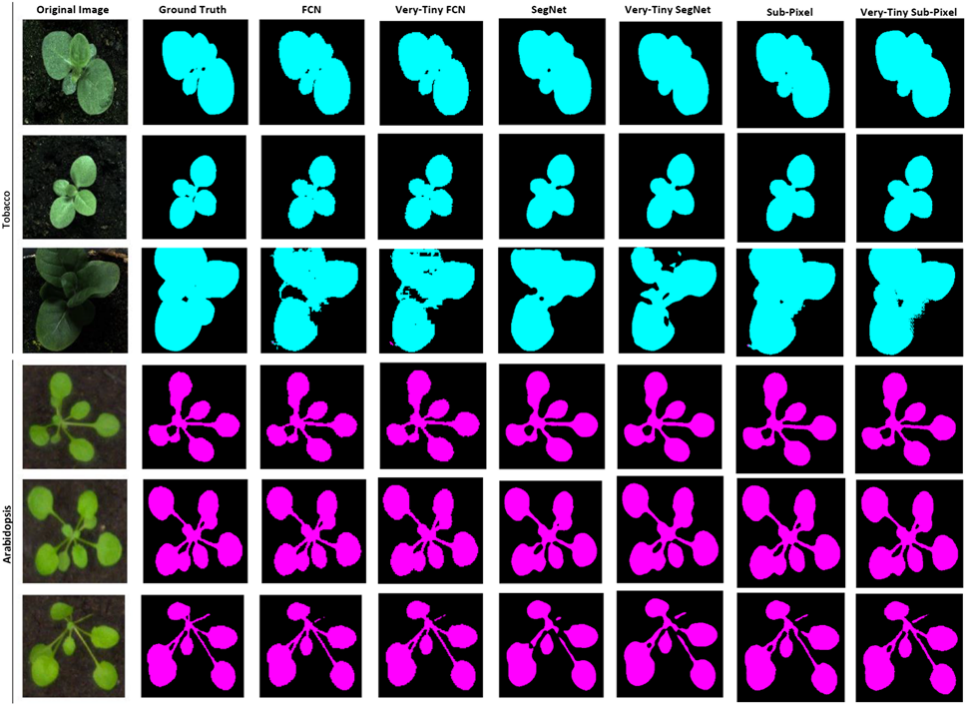
Sample test instances from the Plant Phenotyping dataset

**Fig. 13:**
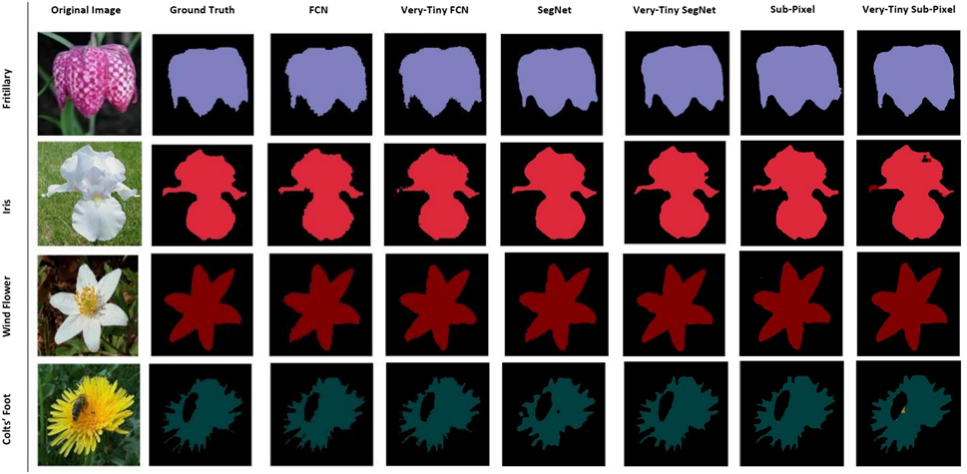
Sample test instances from the Oxford flower dataset with visually very good segmentation (two classes)

**Fig. 14:**
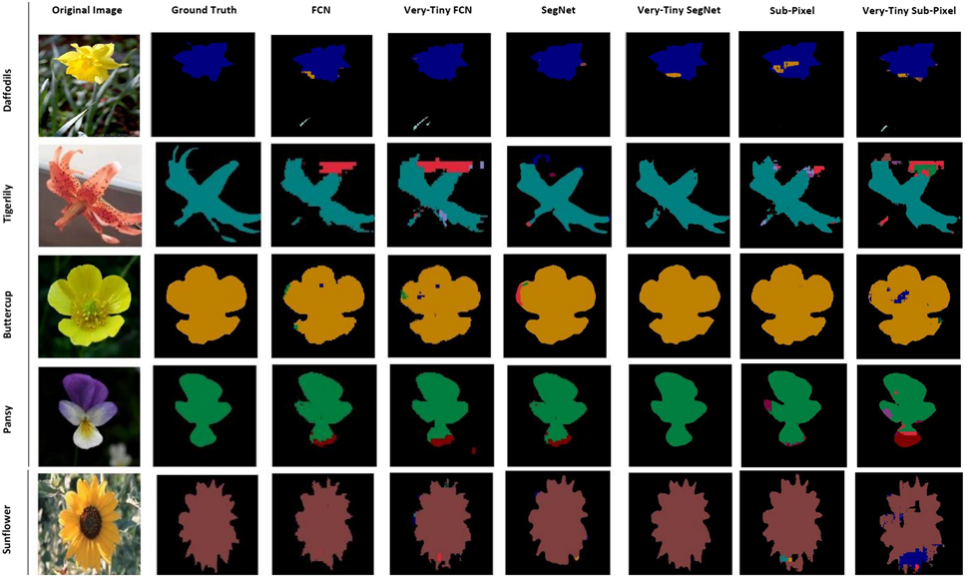
Multi-class segmentation: Sample test instances from the Oxford flower dataset with some segmentation errors present

Our tiny models’ performance on the plant phenotyping datasets compare to their non-compressed counterparts. On test samples where the non-compressed models achieved good segmentation results, the tiny models also did. For example, on the Oxford flower dataset (13-class problem), the Fritillary, Iris, Wind Flower, Colts’ Foot and Daisy test samples in Fig. 13 were well segmented by all models. Additionally, the non-compressed deep CNN counterparts showed better results in some instances than the tiny models and vice versa (see examples in Fig. 14).

**Fig. 15:**
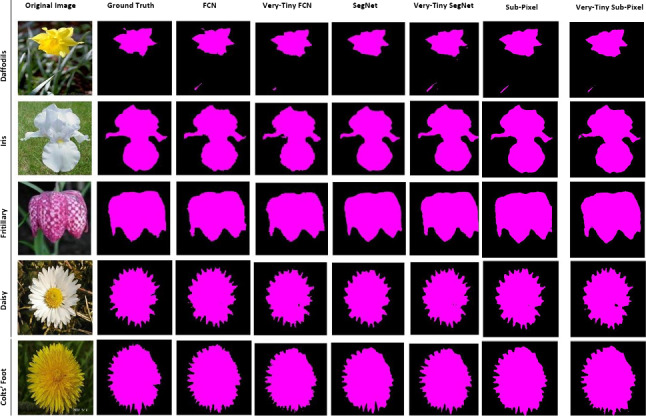
Two-class (background and flower) segmentation: Sample test instances from the Oxford flower dataset with some segmentation errors

**Fig. 16:**
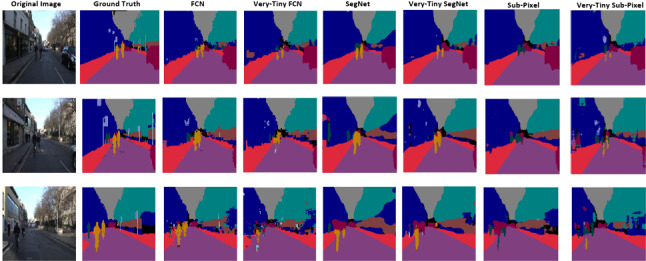
Multi-class segmentation: Sample test instances from the CamVid dataset with some segmentation errors

These observations are true for the plant phenotyping dataset too; see Fig. 12. Using the Oxford flower dataset for background and foreground (flowers) segmentation, the segmentation results of our tiny models were similar to that of their non-compressed deep CNN counterparts (see Fig. 15).

The models with more parameters performed better on the datasets with more classes. For example, SegNet with 17.5 million parameters performed better on the flower and CamVid datasets when compared with all the other models. However, the tiny models’ (Tiny-FCN, Tiny-SegNet and Tiny-Sub-Pixel) performance were comparable to the baseline models on the datasets with fewer classes. The model with the smallest number of parameters is the sub-pixel, which performed better mostly on the datasets with fewer classes. The sub-pixel model’s performance was better on all the datasets except the CamVid, which had more classes. Therefore, truly challenging scenarios may not benefit from the proposed reduction techniques (Fig. 16).

Our tiny models apply the compression technique before model training, which reduces its parameters. Therefore, these models can easily be pre-trained and the pre-trained model loaded and used to initialise another model since they only rely on separable convolutions. Our *very* tiny models, however, cannot be pre-trained as they are compressed after training. The reader interested in pre-training the very tiny models can rather pre-train the tiny models and run the SVD algorithm on the generated weight matrix. It is also important to note that even though our models use the VGG-16 architecture, they cannot benefit from pre-trained models since we have converted the 2D convolutions to 2D separable convolutions.

## Conclusion

We have used two methods (separable convolution and a combination of separable convolution and SVD) to compress three baseline deep CNNs for pixel-wise segmentation. The compressed (tiny) models, when compared to the baselines deep CNN counterpart, obtained more than 88% and 95% parameter reduction and storage space savings, respectively. We have compared our tiny models to some popular compressed models and found that our Tiny-FCN and Tiny-Sub-Pixel were the most compressed models (see Table 8 ). Our Tiny-SegNet models were the fourth most compressed after SqueezeNet.

We evaluated the models on two challenging plant phenotyping datasets (the Oxford flower and plant phenotyping datasets) and a road scene dataset (CamVid). The results from our tiny models were practically as good as their deep CNN counterparts. We noted that where the baseline models classified and segmented plants, flowers and other objects correctly, the tiny models also did in most cases. On plant phenotyping dataset, the Sub-Pixel and FCN models outperformed the SegNet based on mean IoU alone. While the SegNet models were the best on the Oxford flower dataset, we noted a 19% reduction in pixel accuracy when segmenting flowers into 13 classes. Investigations showed that the decrease was due to the inability of baseline deep CNNs (FCN and SegNet) to handle large classes on the plant phenotyping dataset.

Currently, most deep learning approaches are limited to deployment in laboratories due to resource requirements. Ongoing work including ours is aiming to bring these techniques onto low-cost devices for infield plant phenotyping. We have demonstrated the practicality of our tiny models on two mobile devices for infield segmentation of flowers. We noted that on the latest mobile device emulator running the latest Android operating system, it took less than a second to segment flowers, while it took approximately 3.5 s to perform the same task on an old mobile device running a lower version of Android. In the future, we will compress models that are known to perform better on datasets with more classes using our two methods, as an attempt to increase accuracy on the 13-class segmentation problem. We are also working on a cassava root dataset that we wish to release with a benchmark result based on the proposed tiny CNNs introduced in this paper; such a technology will advance phenotyping capability of such crops even in lower- to middle-income countries.

## URL of additional resources

The following resources from this research are available for download from the link in this section’s footnote^2^ :

- All the source code.
- For those not using Python and Keras, the model architectures have been provided in a pdf.
- Model weight matrices including compressed versions
- Graphs of training and validation losses and accuracies against epochs.

## Footnotes

1 https://github.com/Amotica/Low-Cost-Plant-Phenotyping.

2 https://github.com/Amotica/Low-Cost-Plant-Phenotyping.

## References

[1] Aich, S., Stavness, I.: Leaf counting with deep convolutional and deconvolutional networks. (2017) arXiv preprint arXiv:1708.07570

[2] Aich, S., Stavness, I.: Object counting with small datasets of large images. (2018) arXiv preprint arXiv:1805.11123

[3] Aich, S., Josuttes, A., Ovsyannikov, I., Strueby, K., Ahmed, I., Duddu, H.S., Pozniak, C., Shirtliffe, S., Stavness, I.: Deepwheat: Estimating phenotypic traits from crop images with deep learning. In: IEEE Winter Conference on Applications of Computer Vision (WACV), 2018, IEEE, pp 323–332 (2018)

[4] Aich, S., van der Kamp, W., Stavness, I.: Semantic binary segmentation using convolutional networks without decoders. In: 2018 IEEE/CVF Conference on Computer Vision and Pattern Recognition Workshops (CVPRW), IEEE, pp. 182–1824 (2018)

[5] Alexandratos, N., Bruinsma, J. et al.: World agriculture towards 2030/2050: the 2012 revision. Tech. rep., ESA Working paper FAO, Rome (2012)

[6] Atanbori, J., Chen, F., French, A.P., Pridmore, T.: Towards low-cost image-based plant phenotyping using reduced-parameter cnn. In: S A Tsaftaris HS, Pridmore T (eds) Proceedings of the Computer Vision Problems in Plant Phenotyping (CVPPP), BMVA Press, (2018) http://bmvc2018.org/contents/workshops/cvppp2018/0023.pdf

[7] Badrinarayanan V, Kendall A, Cipolla R (2017). Segnet: a deep convolutional encoder-decoder architecture for image segmentation. IEEE Trans. Pattern Anal. Mach. Intell..

[8] Chollet, F.: Xception: Deep learning with depthwise separable convolutions. (2016) arXiv preprint

[9] Denton, E.L., Zaremba, W., Bruna, J., LeCun, Y., Fergus, R.: Exploiting linear structure within convolutional networks for efficient evaluation. In: Advances in neural information processing systems, pp 1269–1277 (2014)

[10] Girshick, R.: Fast r-cnn. (2015) arXiv preprint arXiv:1504.08083

[11] Giuffrida, M.V., Minervini, M., Tsaftaris, S.A.: Learning to count leaves in rosette plants (2016)

[12] Han, S., Mao, H., Dally, W.J.: Deep compression: Compressing deep neural networks with pruning, trained quantization and huffman coding. (2015) arXiv preprint arXiv:1510.00149

[13] Howard, A.G., Zhu, M., Chen, B., Kalenichenko, D., Wang, W., Weyand, T., Andreetto, M., Adam, H.: Mobilenets: Efficient convolutional neural networks for mobile vision applications. (2017) arXiv preprint arXiv:1704.04861

[14] Iandola, F.N., Han, S., Moskewicz, M.W., Ashraf, K., Dally, W.J., Keutzer, K.: Squeezenet: Alexnet-level accuracy with 50x fewer parameters and $$< 0.5~\text{mb}$$ model size. (2016) arXiv preprint arXiv:1602.07360

[15] Jin, J., Dundar, A., Culurciello, E.: Flattened convolutional neural networks for feedforward acceleration. (2014) arXiv preprint arXiv:1412.5474

[16] Jin X, Liu S, Baret F, Hemerlé M, Comar A (2017). Estimates of plant density of wheat crops at emergence from very low altitude uav imagery. Remote Sens. Environ..

[17] Lin, G., Milan, A., Shen, C., Reid, I.: Refinenet: Multi-path refinement networks for high-resolution semantic segmentation. In: IEEE Conference on Computer Vision and Pattern Recognition (CVPR) (2017)

[18] Liu S, Baret F, Andrieu B, Burger P, Hemmerle M (2017). Estimation of wheat plant density at early stages using high resolution imagery. Front. Plant Sci..

[19] Long, J., Shelhamer, E., Darrell, T.: Fully convolutional networks for semantic segmentation. In: Proceedings of the IEEE conference on computer vision and pattern recognition, pp 3431–3440 (2015) 10.1109/TPAMI.2016.2572683 27244717

[20] Minervini, M., Fischbach, A., Scharr, H., Tsaftaris, S.: Plant phenotyping datasets. (2015) http://www.plant-phenotyping.org/datasets

[21] Minervini M, Fischbach A, Scharr H, Tsaftaris SA (2016). Finely-grained annotated datasets for image-based plant phenotyping. Pattern Recogn. Lett..

[22] Minervini, M., Giuffrida, M.V., Tsaftaris, S.A.: An interactive tool for semi-automated leaf annotation (2016)

[23] Nilsback ME, Zisserman A (2010). Delving deeper into the whorl of flower segmentation. Image Vis. Comput..

[24] Pound, M.P., Atkinson, J.A., Townsend, A.J., Wilson, M.H., Griffiths, M., Jackson, A.S., Bulat, A., Tzimiropoulos, G., Wells, D.M., Murchie, E.H., et al.: Deep machine learning provides state-of-the-art performance in image-based plant phenotyping. GigaScience (2017) 10.1093/gigascience/gix083 PMC5632296 29020747

[25] Pound, M.P., Atkinson, J.A., Wells, D.M., Pridmore, T.P., French, A.P.: Deep learning for multi-task plant phenotyping. In: Proceedings of the IEEE Conference on Computer Vision and Pattern Recognition, pp. 2055–2063 (2017)

[26] Razafindradina, H.B., Randriamitantsoa, P.A., Razafindrakoto, N.R.: Image compression with svd: A new quality metric based on energy ratio. (2017) arXiv preprint arXiv:1701.06183

[27] Sandler, M., Howard, A., Zhu, M., Zhmoginov, A., Chen, L.C.: Mobilenetv2: Inverted residuals and linear bottlenecks. In: Proceedings of the IEEE Conference on Computer Vision and Pattern Recognition, pp. 4510–4520 (2018)

[28] Scharr H, Minervini M, French AP, Klukas C, Kramer DM, Liu X, Luengo I, Pape JM, Polder G, Vukadinovic D (2016). Leaf segmentation in plant phenotyping: a collation study. Mach. Vis. Appl..

[29] Shi, W., Caballero, J., Huszár, F., Totz, J., Aitken, A.P., Bishop, R., Rueckert, D., Wang, Z.: Real-time single image and video super-resolution using an efficient sub-pixel convolutional neural network. In: Proceedings of the IEEE Conference on Computer Vision and Pattern Recognition, pp. 1874–1883 (2016)

[30] Shrestha DS, Steward BL (2003). Automatic corn plant population measurement using machine vision. Trans. ASAE.

[31] Sun, Y., Zheng, L., Deng, W., Wang, S.: Svdnet for pedestrian retrieval. (2017) arXiv preprint

[32] Szegedy, C., Liu, W., Jia, Y., Sermanet, P., Reed, S., Anguelov, D., Erhan, D., Vanhoucke, V., Rabinovich, A., et al.: Going deeper with convolutions. Cvpr (2015)

[33] Szegedy, C., Vanhoucke, V., Ioffe, S., Shlens, J., Wojna, Z.: Rethinking the inception architecture for computer vision. In: Proceedings of the IEEE Conference on Computer Vision and Pattern Recognition, pp. 2818–2826 (2016)

[34] Walter A, Liebisch F, Hund A (2015). Plant phenotyping: from bean weighing to image analysis. Plant Methods.

[35] Wang, M., Liu, B., Foroosh, H.: Factorized convolutional neural networks. (2016) CoRR, arXiv:1608.04337

[36] Wu, J., Leng, C., Wang, Y., Hu, Q., Cheng, J.: Quantized convolutional neural networks for mobile devices. In: Proceedings of the IEEE Conference on Computer Vision and Pattern Recognition, pp 4820–4828 (2016)

[37] Xue, J., Li, J., Gong, Y.: Restructuring of deep neural network acoustic models with singular value decomposition. In: Interspeech, pp. 2365–2369 (2013)

[38] Yu, D., Seide, F., Li, G., Deng, L.: Exploiting sparseness in deep neural networks for large vocabulary speech recognition. In: IEEE International Conference on Acoustics, Speech and Signal Processing (ICASSP), 2012, IEEE, pp. 4409–4412 (2012)

[39] Yu, F., Koltun, V.: Multi-scale context aggregation by dilated convolutions. (2015) arXiv preprint arXiv:1511.07122

[40] Yu, F., Koltun, V., Funkhouser, T.: Dilated residual networks. In: Computer Vision and Pattern Recognition, vol 1 (2017)

[41] Zhang, X., Zhou, X., Lin, M., Sun, J.: Shufflenet: An extremely efficient convolutional neural network for mobile devices. (2017) arXiv preprint arXiv:1707.01083

[42] Zhao, H., Shi, J., Qi, X., Wang, X., Jia, J.: Pyramid scene parsing network. In: IEEE Conf. on Computer Vision and Pattern Recognition (CVPR), pp. 2881–2890 (2017)

